# Periradicular Temperature Changes and Risk Management During Heat-Inducing Endodontic Disinfection Procedures In Vitro

**DOI:** 10.3390/jcm14113997

**Published:** 2025-06-05

**Authors:** Theresia Saban, Lea Külzer, Andreas Braun, Johannes-Simon Wenzler

**Affiliations:** Department of Operative Dentistry, Periodontology and Preventive Dentistry, RWTH Aachen University, 52074 Aachen, Germany; lea.kuelzer@rwth-aachen.de (L.K.); anbraun@ukaachen.de (A.B.)

**Keywords:** root canal disinfection, diode laser, sodium hypochlorite, root canal treatment, root surface temperature

## Abstract

**Background/Objectives**: The aim of this study was to investigate the effects of periodontal blood flow on the periapical region during various endodontic disinfection procedures. The hypothesis that periodontal blood flow reduces the increase in root surface temperature during disinfection procedures was tested. **Methods**: One hundred and twenty extracted human teeth were shortened to 11 mm and the root canal was prepared using the F4 ProTaper Gold system. The specimens were covered with wax and then sealed in a thermoforming sheet, leaving a gap of 0.2 mm. Cannulas were attached to simulate stable fluid circulation. Thermographic evaluation was carried out using an infrared camera. The following methods were chosen for disinfection: I, λ445 nm diode laser (0.6 W, cw); II, λ445 nm diode laser, 3 W, pulsed, duty cycle 50%, 10 Hz; III, λ445 nm diode laser, 3 W, pulsed, duty cycle 75%, 10 Hz; IV, λ970 nm diode laser, 2 W, pulsed, duty cycle 50%, 10 Hz; V, λ970 nm diode laser, 2 W, pulsed, duty cycle 75%, 10 Hz; VI, experimental plasma device (2.5 W, 3.7 V); VII, heat plugger (200.0 °C); VIII, NaOCl 3% (60 °C). The results were analyzed statistically using the Kruskal–Wallis test. When there were significant differences between the groups (*p* < 0.05), the pairwise Mann–Whitney test with sequential Bonferroni correction was applied. **Results**: The smallest temperature changes, with a median value of 0.82 °C (max. 2.02 °C, min. 0.15 °C, IQR 0.87 °C), were observed using the laser at a setting of λ445 nm, 0.6 W cw, and a circulation rate of 6 mL/min. The highest temperature changes were measured at a fluid circulation rate of 0 mL/min with a laser setting of λ445 nm, 3 W, pulsed, duty cycle 75% with a median value of 21.7 °C (max. 25.02 °C, min. 20.29 °C, IQR 2.04 °C). **Conclusions**: Disinfection procedures with laser, NaOCl, and an experimental plasma device can lead to an increase in root surface temperature. With the exception of the heat plugger, no significant temperature changes were observed. This study was conducted in vitro, which may limit the direct applicability of the results to clinical scenarios. Nevertheless, the simulation of blood flow showed a thermally protective effect, suggesting that clinical protocols should consider this variable when selecting thermal disinfection methods. These results support the hypothesis that periodontal blood flow may have a potentially positive influence on temperature changes during disinfection procedures.

## 1. Introduction

The success of endodontic treatment depends heavily on the presence of as few residual bacteria as possible in the root canal. These bacteria can enter the tooth through caries or traumatic injuries, for example [1,2]. They migrate to the root apex and cause inflammation of the periapical tissue, which may be radiologically recognizable as apical translucency, an apical lesion [3]. Thorough and effective disinfection of the canal system is crucial to eliminate residual bacteria and ensure the success of endodontic treatment. The studies by Lin et al. and Sjogren et al. support the assumption that complete apical healing can be achieved by eliminating bacteria [4,5]. Insufficient disinfection, however, is associated with subsequent periapical inflammation. In addition, the endodontic treatment should be completed with a tight closure of the canal system. Complete preparation and disinfection of the root canals are prerequisites for obturation. If these criteria are met, the chances of healing are between 74 and 98% [6]. It is essential that the tooth and the surrounding tissue are not subjected to additional stress during endodontic treatment, for example, through thermal influences, as examined in the present study.

The most widely accepted solution used for disinfection is sodium hypochlorite (NaOCl) [7,8,9]. Among the properties ideally exhibited by irrigating solutions are broad antimicrobial activity, inactivation of bacterial endotoxins, and dislodging of pulp and organic and inorganic components of the smear layer [10]. When NaOCl is heated to 60.0 °C, studies have reported further improvement in its disinfection properties [11,12].

Laser technology is an emerging technique with a wide range of applications. Lasers can effectively remove the smear layer and reduce bacteria through thermal, photodynamic, or photothermal methods [13]. Numerous studies have shown that combining disinfecting solutions with laser application significantly reduces bacterial levels [14,15,16]. Laser treatment also leads to a tenfold increase in the penetration depth of disinfectants into dentinal tubules, enhancing decontamination [17,18,19]. The process also seals dental tubules, lowering the risk of tooth reinfection [20,21]. Many studies have proven the penetration of laser energy up to 1000 µm [20,22,23]. The in vivo study by Wenzler et al. [24] showed that a combined application of NaOCl and laser leads to a massive reduction in bacteria in the root canal. In the study mentioned, the number of bacteria was measured both after disinfection with sodium hypochlorite irrigating alone and laser irradiation, and also after combined sodium hypochlorite irrigation and laser irradiation [24].

Another topic, plasma technology, has also shown considerable promise in various fields, particularly in medicine and sterilization. The unique properties of plasma, such as its high reactivity and ability to generate reactive species, make it effective for decontaminating surfaces and inactivating microorganisms, including bacteria, viruses, and even more resilient pathogens such as prions [25,26,27]. The lower temperature requirements (60–70.0 °C) not only protect heat-sensitive materials, but also reduce the risk of thermal damage to equipment. Overall, the versatility of plasma technology is opening up new opportunities for research and applications across various medical fields, enhancing both the efficacy and safety of treatment. The heat inputs created by heated sodium hypochlorite, diode lasers, and also the plasma device have been partly examined in some approaches, but the question arises of the extent to which the energy generated leads to heat development in a real in vitro tooth model. In the present study, we therefore analyzed another established heat source that is used in endodontic therapy, as a comparison group, known as a heat plugger, since it produces a considerable amount of heat and can also be used to heat irrigation fluids in the root canal. The instrument is otherwise routinely used in conjunction with various warm filling techniques for gutta-percha when sealing root canals [28].

The present study investigated the temperature of root dentin during concomitant thermal disinfection procedures and tested the hypothesis that periodontal blood flow reduces heat generation during the application of energy-induced procedures, thereby regulating potential damage.

## 2. Materials and Methods

One hundred and twenty human single-rooted teeth were collected, examined, and stored in physiological saline supplemented with sodium azide (0.9% NaCl, 0.001% NaN_3_). None of the teeth used in the study was extracted for research purposes; the indications for extraction were individual and related to dental care. The study was therefore based on the ethical principles of the World Medical Association Declaration of Helsinki, version 7, 2013) [29] and was approved by the local ethics committee (reference number EK 24-202).

The samples were visually sorted by tooth type and tooth condition. Various single-rooted teeth showing considerable deviations in root length were included.

Teeth were cleaned and shortened with a saw (Primus cut-off grinder, diamond cutter; Walter Messner GmbH, Oststeinbek, Germany) so that the root was only 11 mm long. Based on the extracted teeth available for this study, this provided standardized specimens consisting of root dentin and root cement, but without enamel, to ensure comparability within the study groups. A computer-generated random number table was used to distribute the specimens into individual study groups and to ensure diversity in each test group. The root canals were prepared with hand instruments (Kerr files; Anton Gerl GmbH, Cologne, Germany) up to ISO 25 and mechanically with F1 to F4 ProTaper Gold^®^, ISO size 40/0.06 (Dentsply Sirona Inc., Charlotte, NC, USA), up to 1 mm to patency, analogous to patient treatment. Preparation was performed by irrigation using 5 mL/canal sodium hypochlorite (NaOCl) 2.5% and ethylenediaminetetraacetic acid (EDTA) 17% at 2 mL/canal plus 1 min exposure time, and NaOCl 2.5% at 5 mL/canal for the second disinfection, according to the Aachen endodontic irrigation protocol [30].

To simulate the periodontal gap, the roots were immersed in an immersion wax bath (Dippy Light; Yeti Dentalprodukte GmbH, Engen, Germany, and BEGO^®^ Bremer Goldschlägerei Wilh. Herbst GmbH & Co., Bremen, Germany; temperature 105.0 °C). This created a wax layer 0.2 mm thick. The uniform thickness of the wax layer was checked randomly with a periodontal probe (Carl Martin Gmbh, Solingen, Germany, 973/CP15).

Together with the wax layer, the root was covered with foil (Duran^®^, diameter: 0.5 × 125 mm; Scheu-Dental GmbH, Iserlohn, Germany) in a thermoforming unit (Biostar^®^ from Scheu, Dental GmbH, Iserlohn, Germany). To simulate the 0.2 mm periodontal gap, the wax layer was then removed to create space between the root and the foil. By coloring the wax, the complete and residue-free removal of the wax layer could be optimally monitored. The root was secured to the foil with EvoFlow, so that the simulated periodontal gap was as uniform as possible and the root was not in contact with the foil. Fluid ingress into the root through the apex was prevented by also sealing the apex with EvoFlow (Ivoclar Vivadent, Schaan, Liechtenstein).

Two irrigation cannulas with a capillary tip of 0.48 mm diameter (Ultradent Products GmbH, Cologne, Germany) were used to visualize the fluid circulation in our experiment. These cannulas were integrated into the foil approximately 3 mm above the root apex with Tetric EvoFlow at a precise angle of 45°. The prepared preparation was connected to the tubing system via one of the two cannulas and held in position by a laboratory stand and three-finger clamp.

To enable fluid circulation, the entire model was connected to a pump system (Ismatec^®^, Wertheim, Germany). The setting is made via a digital display, enabling precise dosing and adaptation to respective requirements. The system provides infinitely variable flow rate control, allowing for precise adjustment of the liquid circulation speed and simulation of various test conditions.

This allowed the liquid to circulate constantly through the gap between the cannulas and the model and flow back into the water bath via the second cannula.

**Figure 1:**
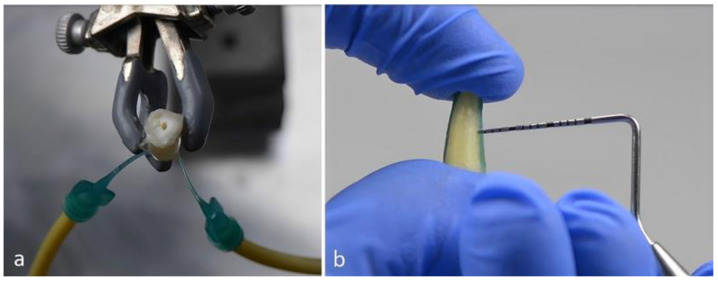
(**a**) Tooth construct, with cannulas and supplying tube system. (**b**) The 0.2 mm wax layer on the tooth preparation.

The system allows the flow rate to be infinitely adjusted, so that the speed of the fluid circulation can be precisely adapted and various experimental conditions can be simulated. Tap water was used for this purpose (Figure 1).

The following study groups were analyzed:

- SiroLaser Blue (λ445 nm, 0.6 W, continuous wave mode, power density 0.00149 W/cm^2^; EasyTip 200 µm Endo, exposure time 4 × 10 s, 5 s pause between laser-activated cycles)
- SiroLaser Blue (λ445 nm, 3 W, pulsed, duty cycle 50%, 10 Hz, power density 0.8 W/cm^2^, EasyTip 200 µm Endo, exposure time 4 × 10 s, 5 s pause between laser-activated cycles)
- SiroLaser Blue (λ445 nm, 3 W, pulsed, duty cycle 75%, 10 Hz, power density 0.8 W/cm^2^, EasyTip 200 µm Endo, exposure time 4 × 10 s, 5 s pause between laser-activated cycles)
- SiroLaser Blue (λ970 nm, 2 W, pulsed, duty cycle 50%, 10 Hz, power density 0.12 W/cm^2^, EasyTip 200 µm Endo, exposure time 4 × 10 s, 5 s pause between laser-activated cycles
- SiroLaser Blue (λ970 nm, 2 W, pulsed, duty cycle 75%, 10 Hz, power density 0.12 W/cm^2^, EasyTip 200 µm Endo, exposure time 4 × 10 s, 5 s pause between laser-activated cycles)
- Wilgoon experimental plasma device (Long-Yao Reisen & Handel GmbH, Leipzig, Germany; intensity level 9, 2.5 W, 3.7 V, power density 0.23 W/cm^2^; 850 mAh lithium polymer battery; cannula (B. Braun Sterican^®^, Melsungen, Germany, standard cannula, Ø 0.80 × 40 mm), exposure time 4 × 10 s, paused 5 s)
- Heat plugger (BeeFill 2in1 device (VDW Dental, Munich, Germany), heated to 200.0 °C, exposure time 4 × 10 s, paused 5 s)
- Sodium hypochlorite 3% (60.0 °C, heated by a thermal bath, exposure time 60 s)

During the application of the respective systems, a thermal imaging camera (VarioCAM HD; InfraTec^®^ GmbH, Infrarotsensorik und Messtechnik, Dresden, Germany) was used to record heat development. Images were acquired at 1 s intervals for a total duration of 1 min 20 s and displayed on the connected laptop using IRBIS 3 plus software (InfraTec GmbH Infrarotsensorik und Messtechnik, Dresden, Germany).

The simulated sulcus fluid was heated to a physiological temperature of 36.0–37.0 °C using a heating plate (VMR Advanced, VWR international, Radnor, PA, USA) and magnetic stirrer (Fisher Scientific GmbH, Schwerte, Germany). At the flow rates of 2.6 mL/min and 6 mL/min with the heated sulcus fluid, the temperature in the simulated periodontal crevice was kept constant. In the 0 mL/min experiments, the specimens were preheated to a temperature of 37.0 °C on the tooth. Temperature control during the entire test was continuously monitored and controlled using a thermal imaging camera and IRBIS 3 plus software (InfraTec, Dresden, Germany). This combination enabled precise recording and monitoring of the temperature curves and temperature changes on the surface of the samples. This ensured reliable control of the temperature throughout the experiment and helped to keep the conditions constant to ensure the validity and accuracy of the measurement results.

For the laser procedures in groups I to V, the SiroLaser Blue with an EasyTip Endo (SiroLaser; Dentsply Sirona, Germany GmbH, Bensheim, Germany) was used. The laser was used at wavelengths of 445 nm and 970 nm in this study. The radiation mode can be selected between pulse mode and continuous-wave mode.

The laser with the 0.6 W setting was taken as the comparison group. The EasyTip 200 µm Endo served as the light guide. The fiber was angled slightly with the aid of a manufacturer-specific bending tool, resulting in an angle of approximately 45°. This simplified the working method. NaOCl was poured into the root canals. During implementation in all laser groups, the fiber was introduced up to 1 mm in front of the apex and guided in helical movements from the apical to coronal part of the root (1 mm/s). In this case, the laser time was 10 s. The procedure was repeated four times.

The plasma device in group VI (WILGOON, Long-Yao Reisen & Handel GmbH, Leipzig, Germany) was clamped to a cannula (B. Braun Sterican^®^ standard cannula, Ø 0.80 × 40 mm) at the tip. This made it possible to choose between the different intensity levels 1–9 (0.4–2.5 W). Level 9 was selected for this study, corresponding to 2.5 W. Again, the plasma device was moved in helical movements from the apical to coronal part of the root (1 mm/s). This process was repeated several times.

The heat plugger in group VII, with the BeeFill 2in1 device (VDW Dental, Munich, Germany) was adjusted to 200.0 °C and, like the laser tip, was led to 1 mm in front of the root tip and moved up and down in spiral motions from the apical to the coronal part of the root (1 mm/s).

For standard disinfection with NaOCl 3% (group VIII), the solution was drawn up into 10 mL syringes and heated in hot water to 60.0 °C. Subsequently, the root canals were rinsed with the heated NaOCl during the entire uptake time.

**Figure 2:**
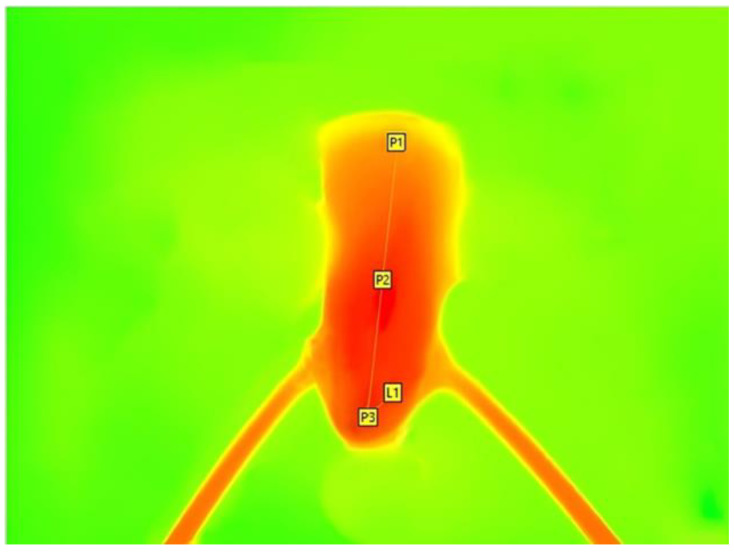
Temperature measurement during obturation, performed with a thermal imaging camera (VarioCAM HD, InfraTec GmbH Infrarotsensorik und Messtechnik, Dresden, Germany) at measurement points P1, P2, and P3, set at the coronal, middle and apical third, with the L1 line indicated.

After completion of the experiment, heat generation in the simulated periodontal gap was detected using the three individual measurement points P1, P2, and P3 and a line L1, which is the range of measurement (Figure 2). The line connects the three measurement points. Both the measurement points and the measurement range were set from coronal to apical, parallel to the tooth axis.

**Figure 3:**
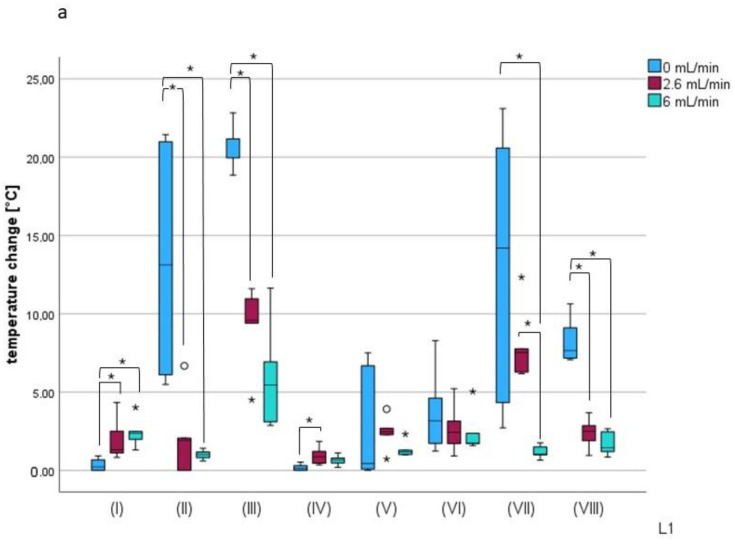

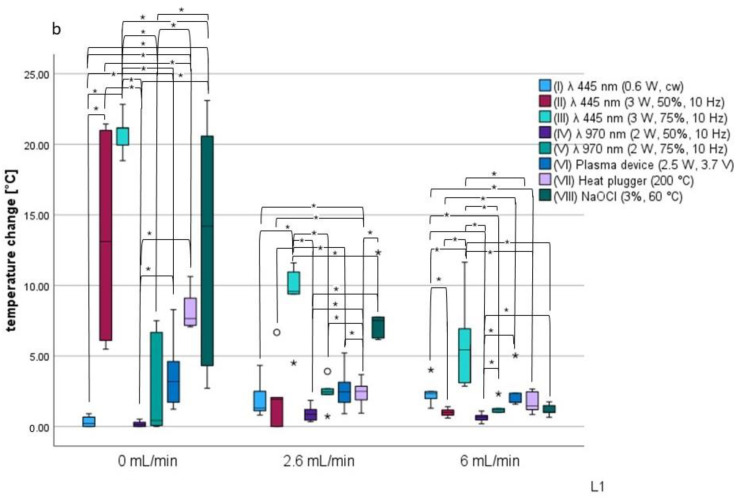
(**a**) Box plots for temperature changes at L1 at different fluid circulation rates, as a function of the disinfection technique. (**b**) Box plots for temperature changes at L1, showing mean values for all root sections in the thermographic images. Temperature changes in the disinfection techniques are compared as a function of the fluid circulation rate. Asterisks (*) between box plots represent a *p* value < 0.05. ◦ represents outliers 1.5 to three times the interquartile range; asterisks above the boxplots represent distant outliers.

The sequences recorded were converted into a temperature–time diagram using the IRBIS 3 Plus software program. The initial temperature of the tooth (T_0_), the maximum temperature reached (T_max_), the latency time to reach the maximum temperature, and the delta between T_max_ and T_0_ were included in the analysis. The data were checked for normal distribution (Shapiro–Wilk test). Since data were not normally distributed and the study involved multiple comparison, statistical analysis was performed using the Kruskal–Wallis test. If the values showed a significant difference at *p* < 0.05, the Mann–Whitney pairwise test with sequential Bonferroni correction was applied. The median, first, and third quartiles, as well as the minimum and maximum values, are shown in the box plot diagrams (Figure 3a). Values that exceeded the interquartile range by one and a half to three times are indicated as outliers, and appear with a data point in the diagrams. Values exceeding more than three times the interquartile range are marked with asterisks, as remote outliers.

## 3. Results

### 3.1. Temperature Changes with Disinfection Techniques Relative to Fluid Circulation Rate (L1)

The first group represented temperature changes with the different disinfection options without liquid circulation. Significant temperature differences were found between the different techniques (*p* < 0.05). At 0 mL/min in the graph, it can be seen that in group III, there is a significantly high temperature excursion. The median value here was 21.15 °C (max. 22.83 °C, min. 18.84 °C, IQR 1.21 °C). The lowest temperature difference occurred in group IV, where the median value was 0.14 °C (max. 0.52 °C, min. 0.0 °C, IQR 0.3 °C) (Figure 3a, Table 1).

The largest increase at 2.6 mL/min was again in group III, recorded with a median value of 9.57 °C (max. 11.6 °C, min. 4.5 °C, IQR 1.56 °C). There was a significant difference between the sample medians (*p* < 0.05). Group IV showed the lowest heat generation, with a median value of 0.87 °C (max. 1.85 °C, min. 0.35 °C, IQR 0.76 °C) (Figure 3a, Table 1).

There was also a temperature difference at 6 mL/min (*p* < 0.05). The highest value, with a median of 5.44 °C (max. 11.64 °C, min. 2.86 °C, IQR 3.83 °C) was again seen in group III, at 445 nm with a duty cycle of 75%. The lowest value here was measured in group IV, with a median value of 0.67 °C (max. 1.1 °C, min. 0.19 °C, IQR 0.31 °C) (Figure 3a, Table 1).

### 3.2. Temperature Changes at Different Fluid Circulation Rates Relative to Disinfection Techniques (L1)

In group I, the largest difference from the initial temperature was seen at 6 mL/min, with a median value of 2.35 °C (max. 4.01 °C, min. 1.3 °C, IQR 0.5 °C). The tooth was heated least at 0 mL/min. The median here was 0.22 °C (max. 0.91 °C, min. 0 °C, IQR 0.67 °C). The three flow rates all showed temperature differences, so that a significant difference in temperature change was observed (*p* < 0.05) (Figure 3b, Table 1).

The technique used in group II showed the largest temperature excursion at 0 mL/min, with a median value of 13.11 °C (max. 21.44 °C, min. 5.48 °C, IQR 14.88 °C). The lowest value was seen at 6 mL/min, with a median of 1.01 °C (max. 1.41 °C, min. 0.6 °C, IQR 0.36 °C) (Figure 3b, Table 1).

In group III, the largest temperature differences were also found at 0 mL/min, at 21.15 °C (max. 22.83 °C, min. 18.84 °C, IQR 1.21 °C) and 2.6 mL/min 9.57 °C (max. 11.6 °C, min. 4.5 °C, IQR 1.56 °C). At 6 mL/min, the median value was 5.44 °C (max. 11.64 °C, min. 2.86 °C, IQR 3.83 °C). Striking differences were recognizable in both the 50% and 75% duty cycles (*p* < 0.05) (Figure 3b, Table 1).

In group IV, the median value at 2.6 mL/min was 0.87 °C (max. 1.85 °C, min. 0.35 °C, IQR 0.76 °C). The temperature difference was greatest here. The circulation rate of 0 mL/min showed the lowest increase in temperature, with a median of 0.14 °C (max. 0.52 °C, min. 0 °C, IQR 0.3 °C) (Figure 3b, Table 1).

The only higher temperature in group V was observed at 2.6 mL/min, with a median of 2.46 °C (max. 3.91 °C, min. 0.72 °C, IQR 1.81 °C), and the lowest increase was seen at 6 mL/min, with a median of 1.17 °C (max. 2.31 °C, min. 0.99 °C, IQR 0.29 °C). However, both methods showed a significant difference between the three flow rates (*p* < 0.05) (Figure 3b, Table 1).

With the plasma device in group VI, a median value of 3.17 °C (max. 8.28 °C, min. 1.23 °C, IQR 2.89 °C) was measured at 0 mL/min; a median of 1.73 °C (max. 5.03 °C, min. 1.58 °C, IQR 0.65 °C) was measured at 6 mL/min. There were significant temperature differences (*p* < 0.05) (Figure 3b, Table 1).

The largest temperature increase was also seen at 0 mL/min in group VII. The median value here was 14.2 °C (max. 23.1 °C, min. 2.71 °C, IQR 16.26 °C). Comparison of the flow rates within this application also revealed a difference in the medians of the individual specimens (*p* < 0.05). The lowest difference was at 6 mL/min, with a median value of 1.02 °C (max. 1.75 °C, min. 0.65 °C, IQR 0.5 °C) (Figure 3b, Table 1).

When the teeth in group VIII were rinsed with NaOCl heated to 60 °C, the temperature increase was found to be highest at 0 mL/min, with a median value of 7.65 °C (max. 10.63 °C, min. 7.06 °C, IQR 1.93 °C), compared to 6 mL/min, with a median value of 1.45 °C (max. 2.66 °C, min. 0.85 °C, IQR 1.26 °C). Again, differences between the median values were evident (*p* < 0.05) (Figure 3b, Table 1).

### 3.3. Temperature Changes in the Coronal Third of the Root (P1)

**Figure 4:**
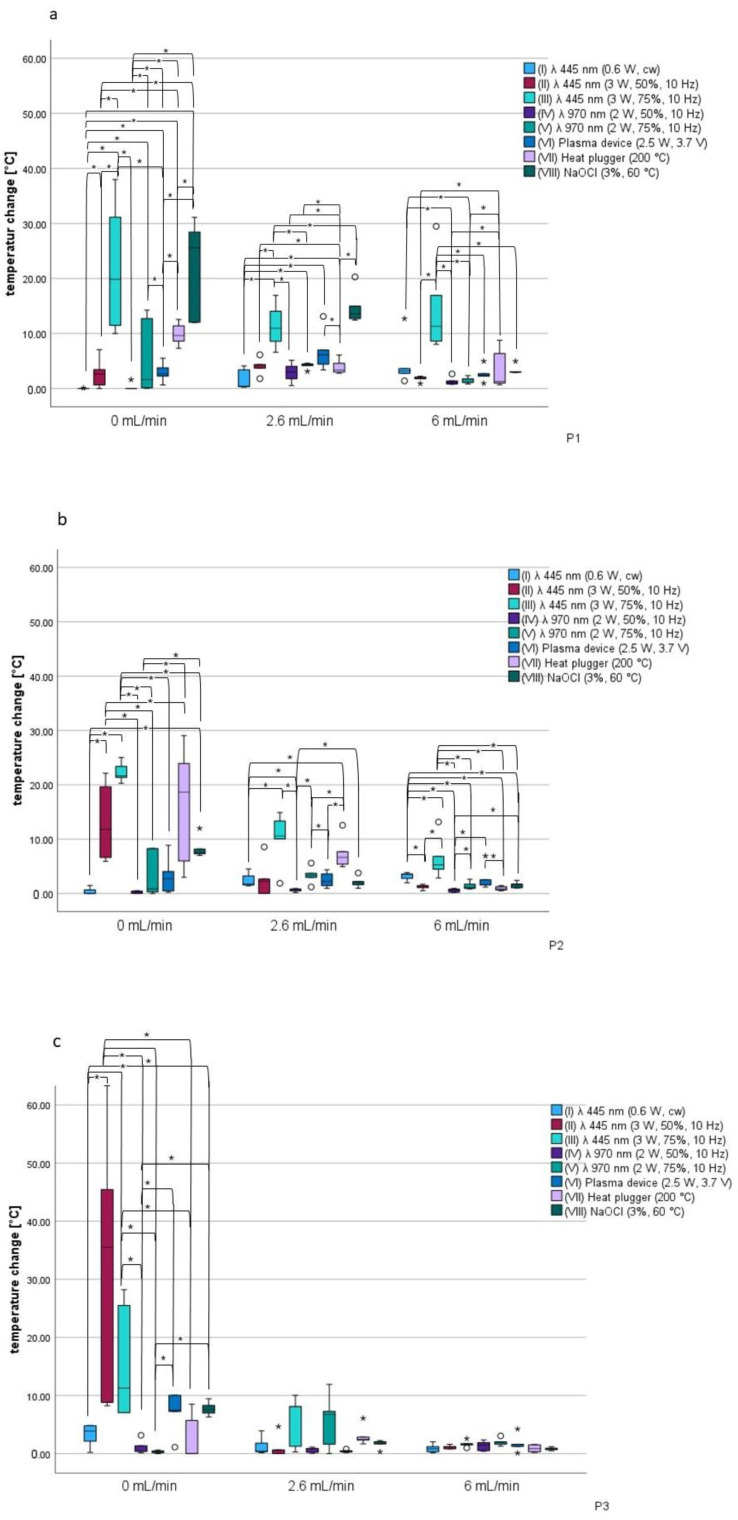
(**a**) Box plots for P1: temperature changes in the coronal third relative to the fluid circulation rate. (**b**) Box plots for P2: temperature changes in the middle third of the root relative to the fluid circulation rate. (**c**) Box plots for P3: temperature changes in the apical third relative to the fluid circulation rate. Asterisks (*) between the box plots represent a *p* value < 0.05. ◦ represents outliers 1.5 to three times the interquartile range; asterisks above the boxplots represent distant outliers.

Temperature changes in the coronal third of the root were assessed at measurement point P1. There were significant differences (*p* < 0.05). The highest temperature at 0 mL/min was found in group VII, with a median value of 25.58 °C (max. 31.12 °C, min. 12.01 °C, IQR 16.39 °C). The lowest temperature, by contrast, was seen in group I, also at 0 mL/min, with a median value of 0.00 °C (max. 0.11 °C, min. 0.00 °C, IQR 0.00 °C) (Figure 4a, Table 2).

At a circulation rate of 2.6 mL/min, the highest temperature changes were again in group VII, with a median value of 13.53 °C (max. 20.28 °C, min. 12.45 °C, IQR 2.26 °C) and in group III, with a median value of 10.94 °C (max. 16.95 °C, min. 6.59 °C, IQR 2.26 °C). The lowest was found in group I with a median value of 0.4 °C (max. 4.1 °C, min. 0.26 °C, IQR 3.07 °C) (Figure 4a, Table 2). There was a significant difference between the medians for the specimens (*p* < 0.05).

Group III showed the highest temperature change at 6 mL/min, with a median value of 11.28 °C (max. 29.47 °C, min. 0.9 °C, IQR 0.28 °C), while the lowest temperature change was noted in group IV, with a median value of 1.1 °C (max. 2.65 °C, min. 0.75 °C, IQR 0.54 °C) (Figure 4a, Table 2). The sample medians were significantly different (*p* > 0.05).

### 3.4. Temperature Changes in the Coronal Third of the Root (P2)

The middle section of the root was labeled P2. The highest overall temperature was measured here for group III at 0 mL/min, with a median value of 21.7 °C (max. 25.02 °C, min. 20.29 °C, IQR 2.04 °C). Group I stood out with a median value of 0.03 °C at 0 mL/min for the lowest temperature (max. 1.5 °C, min. 0.00 °C, IQR 0.68 °C) (Figure 4b, Table 2) Again, differences between the medians were apparent (*p* < 0.05).

At a flow rate of 2.6 mL/min, group III again had the highest temperature change, with a median value of 10.6 °C (max. 14.92 °C, min. 1.89 °C, IQR 3.29 °C). The lowest temperature change was observed in group IV, with a median value of 0.54 °C (max. 0.92 °C, min. 0.19 °C, IQR 0.36 °C) (Figure 4b, Table 2). The median differences were also found to differ (*p* < 0.05).

Group III showed a high temperature change at 6 mL/min, with a median value of 5.3 °C (max. 13.16 °C, min. 2.84 °C, IQR 2.34 °C), and the lowest change was noted in group IV, with a median value of 0.67 °C (max. 0.93 °C, min. 0.2 °C, IQR 0.53 °C) (Figure 4b, Table 2).

There was a significant difference between the sample medians (*p* < 0.05).

### 3.5. Temperature Changes in the Coronal Third of the Root (P3)

Temperature changes in the apical third of the root were visualized at P3. The sample medians differed significantly (*p* < 0.05). With a median value of 35.52 °C (max. 63.31 °C, min. 8.23 °C, IQR 36.63 °C), group II showed the highest temperature change at 0 mL/min. The lowest temperature was observed in group VII, with a median value of 0.03 °C (max. 8.51 °C, min. 0 °C, IQR 5.7 °C) at 0 mL/min (Figure 4c, Table 2).

At a flow rate of 2.6 mL/min, the highest temperature change was observed in group III, with a median value of 8.06 °C (max. 10.03 °C, min. 0.28 °C, IQR 6.85 °C), while the lowest change was observed in group VI, with a median value of 0.41 °C (max. 0.79 °C, min. 0.17 °C, IQR 0.2 °C) (Figure 4c, Table 2). There were no significant differences between the sample medians (*p* > 0.05).

The highest temperature change at 6 mL/min was measured with a median value of 1.87 °C (max. 3.05 °C, min. 1.27 °C, IQR 1.2 °C) in group V. The lowest temperature change measured was in group I, with a median value of 0.82 °C (max. 2.02 °C, min. 0.15 °C, IQR 0.87 °C). There were no significant differences between the medians of the samples (*p* > 0.05) (Figure 4c, Table 2).

## 4. Discussion

Reducing bacteria is one of the positive properties of using lasers during endodontic treatment. In a pilot study, Katalinić et al. showed a reduction in three bacteria found in the root canal—*Enterococcus faecalis*, *Staphylococcus aureus*, and *Candida albicans*—during irradiation at wavelengths of 445 nm and 970 nm [31]. The same laser settings were also chosen for the present study.

Studies show that tissue absorbs laser light largely based on wavelength. Shorter wavelengths, such as 445 nm, are absorbed more strongly, especially by oxygen-containing tissue, which leads to increased heat generation. If not controlled, this increased absorption can lead to more intense thermal effects, resulting in tissue damage or undesirable side effects. In contrast, longer wavelengths, such as 970 nm, are absorbed less by oxygen-containing tissue, resulting in a potentially safer application. Therefore, the choice of laser parameters, especially the wavelength, is key to achieving the desired treatment results while minimizing the risk of tissue damage [32].

The side effects of laser use, such as a localized increase in temperature, are being investigated [33]. A study of animal bones by Eriksson and Albrektsson reports that even heating to 47.0 °C threatens the critical limit for bone survival [34]. Another study in connection with obturation techniques discusses whether the cementoblasts located in the periodontal gap develop a lower mRNA expression of mineralized tissue-associated proteins due to heat exposure. Reduced expression might impair apical healing [35].

R. Livada et al. show that thermal removal of gutta-percha during endodontic treatment can lead to bone necrosis [36]. Even low temperatures can cause cellular changes. In human periodontal ligament fibroblasts, exposure to heat leads to altered expression of RANKL and OPG, which affects bone remodeling [37]. A study by Lau et al. shows that dental pulp stem cells respond to heat stress; a temperature increase of 5.5 °C can cause irreversible damage to the vital dental pulp [38]. Although this specific issue was not addressed in the present study, it illustrates the temperature sensitivity of these cells. Periodontal ligament cells produce heat-shock proteins in response to various stressors, which may play a role in the development of periodontitis [39]. This illustrates the complexity of the interactions between thermal effects and biological responses.

When analyzing the data, some values were identified as outliers, significantly different from the other measured values. These extreme values could be due to methodological errors, uneven conditions during the measurement, or special physiological conditions. For example, a maximum value of 63.31 °C (median value of 35.52 °C) was found for the laser application in group II. Such deviations may indicate technical errors, for example. Possible causes could be laser positioning or thermography errors.

Incorrect alignment of the laser can lead to uneven heat distribution and thus to excessive temperature values [40]. Thermography errors should also be considered, as inaccurate measurements can significantly bias the results.

The anatomy of the tooth may also play a role. If a tooth has atypical anatomy, such as thinner apical dentin, this could affect heat conduction and distribution in the tissue, leading to higher temperature readings [41]. This was only checked visually when selecting the tooth samples. Such anatomical variations are difficult to assess in practice. From a clinical point of view, there is an increased risk of tissue damage at these high temperatures, for example, due to lack of blood flow due to systemic diseases.

In order not to distort the significance of the results, the analysis examined whether the exclusion of these discarded data influenced the results. It was found that the results remained stable even without the extreme values, indicating that the data collected are reliable. For a more accurate analysis of temperature variations, it would be useful to use additional temperature measurement points. This would not only provide a more detailed picture of the thermal effects of the treatment, but would also help to identify possible overheating risks in specific areas. This would increase the validity of the results and provide useful information for practical application. Therefore, it would be useful to include additional temperature measurement points in future studies. For even more accurate temperature measurement, future approaches could also consider a combination of additional temperature measuring points and thermocouples.

It is believed that periodontal blood flow may help reduce the heat [42]. In the present study, three different fluid circulation settings were chosen. The calculation of fluid movements of 0 mL/min, 2.6 mL/min, and 6 mL/min in the periodontal gap is based on studies by Birn and by Walling et al. [43,44]. The five teeth per test group were selected according to the study by Walling et al. [44] and an associated power analysis. During inflammation, blood flow to the tissue is increased (6 mL/min) [45]. In contrast, individuals who smoke [46] and those with diabetes [47] tend to have decreased blood flow (2.6 mL/min). No blood flow at all corresponds to 0 mL/min.

The highest surface temperature at the root in the study by Da Fonseca Alvarez et al. [48] was achieved with the 3.5 W laser setting, which was more than 7.0 °C above the initial temperature. Da Fonseca Alvarez et al. dried the root canal before using the laser, guided the laser outward in circular movements over the canal wall, and allowed the tooth to cool for 20 s [48]. In the present case, however, the NaOCl solution is left in the root canal. The higher values observed in the study could be due to the continuous use of the laser for the entire exposure time of 1 min. The values in the study by Da Fonseca Alvarez et al. [48] are comparable to our results, and the presence of NaOCl in the canal may therefore not appear to be a decisive factor.

At a flow rate of 0 mL/min, significant temperature fluctuations were observed when the diode laser was operated with a wavelength of 445 nm and a duty cycle of 50% and 75%. This can be explained by the pulsed laser beam. The total energy during the application is the cumulative total of the individual energy pulses and is thus potentiated [32]. It should be remembered that the absorption spectra of the wavelengths 445 nm and 970 nm differ; 970 nm is better absorbed in water, and the energy may be converted into heat. The individual setting of the duty cycle can also lead to differences. The fact that a duty cycle of 75% results in a significantly higher value than the duty cycle of 50% in this comparison may be due to other variables, such as the amount of sodium hypochlorite in the canal, or also the dwell time of the laser tip in the apical portion of the root. Thus, the maximum average temperature of 22.83 °C at 445 nm with a 75% duty cycle was reached only after a latency of 61.07 s. With the same duty cycle but 970 nm, a maximum average temperature of only 7.5 °C was measured after a latency time of 64.88 s.

The question thus arises of the extent to which the environment influences the temperature measurements. The simulated flow rate was used in an attempt to keep the temperature in the tooth at 37.0 °C, at least approximately. A recent study by Stănuși et al. [49] used a virtual three-dimensional model of a molar in an attempt to mimic physiological oral conditions as closely as possible. A finite element analysis (FEA) program was used to record the temperature increase in both the root and the surrounding periodontal tissue during treatment with a diode laser [49]. Although the results showed very high temperatures—up to 400 °C—that were maintained for less than 0.5 s on the inner walls of the root, the temperature measurements in the periodontal area remained consistently within physiological levels. However, the extent to which the results are comparable with ours is questionable, as the finite element analysis study did not involve a real tooth model. In contrast to the present study, in which specific points were selected for heat measurement, FEA includes heating of the entire surface. In the present study, specific points were used to measure the temperature, which may indeed lead to a limited view. In addition, the use of approximation methods in FEA can lead to some inaccuracy, especially when dealing with complex biological systems. FEA is based on the assumption of linearity. This is a major limitation, as heat transfer in biological tissues is often nonlinear. A major advantage of the present study is that the heat development was recorded in real time using a thermal imaging camera (VarioCAM HD; InfraTec GmbH, Dresden, Germany). This technology allows immediate detection of temperature changes and can help adjust treatment techniques in real time to minimize the risk of thermal injury.

However, Braun et al. report that long-term use of the laser at 970 nm increases the development of microcracks in the dentin [50]. This raises the question of whether the positive feature of little or no heating of the periapical tissue outweighs the weakening of the tooth by the laser. Braun et al. [50] recommend strict adherence to the laser protocol in order to prevent these side effects; but their study also shows that the resulting cracks are comparable to the cracks after calcium hydroxide deposition.

In a study by Beer et al., the temperature difference at the root surface was investigated using a laser setting with a wavelength of 980 nm [51]. The results show a maximum of +5.7 °C temperature increase on the root surface during treatment. It should be mentioned that in the study by Beer et al. [51], the laser was held in the canal for a maximum of 5 s and then paused for 20 s. This allows the root surface to cool down during laser application. Although different watt settings were examined in the study by Beer et al. [51], the duty cycle was the same in the groups examined. In contrast to the present study, the results therefore showed no significant differences. In the present study, the laser application time was longer (4 × 10 s) and the cooling time shorter (5 s) than in Beer et al. [51]. This could explain the higher temperature measurements. The settings were taken from the in vivo study by Wenzler et al. in order to investigate the clinically applied parameters in vitro with regard to heat development [24].

This allows the maximum effects of laser treatment to be evaluated under controlled conditions. Such an approach allows observation of the immediate effects of laser energy on the tissue without the variability that can result from longer cooling intervals. The longer application times, compared to those in the referenced study, represent an extreme in terms of heat generation.

The temperatures generated in the present study remained mostly below the critical value for periodontal tissue [35,37,39]. The few instances of excess can be attributed to the duration of the application.

The plasma device method is not yet widely used for disinfecting root canals. Studies with plasma have confirmed that it has a bactericidal effect, which could also contribute to the disinfection of the root canal in endodontics [52,53]. In this study, the highest possible device setting was selected. It is assumed that this setting achieves the best possible disinfection of the root canal.

In the experiments, it was shown that the temperature in the periapical tissue remained constant. The cooling effect of the periapical fluid is clearly recognizable when one looks at the individual values for P1–3 in the respective flow rates. The lowest temperature differences can be seen in the values for P3. In addition to the slight increase in temperature in this study, Eggers et al. reported another positive effect [54]. They examined the mineralization and cell proliferation of dental cementoblasts and found that they were stimulated by the treatment [54]. These positive effects on regeneration-associated cell functions could support the apical healing process and thus the healing of root diseases. It is questionable whether the use of pen disinfection outweighs the effects of the laser. Using the laser in combination with sodium hypochlorite has led to a significant reduction in bacteria in some studies [17,18,19]. Further studies will need to examine whether the reduction in bacteria using the plasma device is comparable to the reduction achieved with laser disinfection. Recent investigations have already focused on the effect of atmospheric plasma on human osteoblasts [55,56].

In the present study, the heat plugger was not used in connection with gutta-percha, but represents a method comparable with lasers, plasma devices, and NaOCl with regard to temperature and the associated potential periodontal damage. In a study by Walters and Rawal, the damage to the periodontal ligament and alveolar bone after prolonged exposure to heat was so severe that ankylosis was observed in the alveolar region after one month. Only ultrasonic devices were used here [57]. While the highest temperature changes were observed in the middle third, our study showed stronger temperature changes in the coronal third of the root or at 2.6 mL/min or 0 mL/min. The high temperatures can be explained by the fact that the heat plugger was continuously moved up and down in the root canal, there was no thermal insulation of gutta-percha [58] and, of course, the flow rates were lower. An in vitro study by Külzer et al. [28] also investigated temperature effects on apical tissue in relation to periodontal flow rates. Different obturation techniques were investigated, including the use of the heat plugger in two test groups. In the study by Külzer et al., no critical temperature increases were observed [28]. On the basis of our data, therefore, the heat plugger as a comparison group therefore does not show any potentially harmful influence, even in connection with the heating of irrigating solutions.

Furthermore, NaOCl heated to 60.0 °C was used as a further disinfection option. Some studies have shown that heating NaOCl further enhances its properties [11,12], especially the tissue-dissolving effect [10]. At the same time, heating does not increase toxicity [59]. However, heating to 60.0 °C increases the antibacterial effect on microorganisms [60,61]. Due to the slow and continuous flushing of the canal, it is reasonable to assume that this could also heat the periapical tissue. A direct comparison of the NaOCl with the laser settings examined shows that heated NaOCl hardly causes a significant temperature increase in the tissue. A study by Yared et al. [61] suggests that heating NaOCl is an effective adjunct to root canal irrigation for improving disinfection in apical lesions. The results showed that the samples were free of bacteria when the heated NaOCl method was used, whereas the conventional irrigation method was less effective [61].

This is an advantage of the chemical disinfection effect. The temperature increases observed in group VIII in this study remained at levels that are not considered critical for periodontal tissue due to the simulated blood flow.

It is important to note that this in vitro study has some limitations that should be considered. Only single-rooted teeth were included in the present study, and areas with reduced dentin thickness, such as the furcation zones in multi-rooted teeth, were not included. Additionally, adjunctive disinfection methods are becoming more common in clinical practice, raising concerns about potential thermal damage to periodontal tissue.

Finally, the question arises as to what extent the tested disinfectants are comparable in terms of their bactericidal mode of action. Laser and plasma technologies offer an extremely effective bactericidal effect, as they are able to kill bacteria and toxins by emitting intense energy [49]. The improved penetration depth into the dentin tubules is a clear advantage over purely mechanical or chemical disinfection methods. As our study has shown, thermal safety is an important aspect, as the use of laser and plasma technologies is associated with potential heat generation. Finding the right balance is important here. The energy must be high enough to achieve a bactericidal effect, yet low enough to avoid damaging the surrounding tissue or causing undesirable thermal side effects. Kivanc et al. [62], for example, have addressed this issue. Their study showed that diode lasers are effective at eliminating E. faecalis in root canals, regardless of power (1.2 W, 2 W, or 3 W). While the antimicrobial effect was comparable, the higher power resulted in significantly greater temperature increases on the root surface, similar to our study [62]. Unfortunately, research into the application of plasma technology in endodontics is still limited, so further studies are needed to determine specific safety parameters and ensure effective and safe procedures.

NaOCl is the most commonly used chemical disinfectant in endodontic therapy because it effectively kills bacteria, dissolves biofilms and disinfects the remaining structures. However, there are certain aspects to consider when using NaOCl: improper use can result in tissue etching, especially if the solution leaks into the surrounding tissue. This can lead to pain, swelling, and other complications. Therefore, NaOCl is essential for disinfection and should therefore be applied precisely to ensure optimum disinfection performance without damaging the surrounding tissue [63].

Combining chemical disinfection with NaOCl and physical disinfection with a laser or plasma can significantly increase treatment effectiveness. Although sodium hypochlorite is primarily used for the chemical disinfection of root canals, laser and plasma technologies effectively kill microorganisms due to their ability to penetrate deeply, even in hard-to-reach areas [24]. Sarda et al. [64] conducted a study investigating the effectiveness of diode lasers and photoactivated disinfection in combination with NaOCl on E. faecalis and S. mutans. The results showed that using NaOCl with either diode laser or photoactivated disinfection significantly reduced the bacterial load [64].

In an in vitro study, Katalinic et al. [65] investigated the effectiveness of a 445 nm diode laser in enhancing the antimicrobial effect of 2.5% NaOCl. Additionally, the researchers compared it to an established 970 nm diode laser protocol to evaluate the efficacy of both procedures in eliminating root canal pathogens. Results showed that the 445 nm NaOCl irradiation protocol exhibited significantly improved antimicrobial efficacy compared to NaOCl irrigation alone. In ex vivo experiments, it also showed comparable efficacy to the 970 nm protocol [65].

The results of the present study show that critical temperature increases of more than one minute do not occur when using different disinfection techniques and considering periodontal blood flow.

Due to their controlled heat development, these supplementary thermal disinfection procedures can be used without hesitation. They significantly reduce the bacterial load in the root canal, thus probably increasing the success of endodontic treatments. Careful implementation of the procedures is essential to ensure safe and effective application.

## 5. Conclusions

The use of heat-inducing procedures in endodontic treatment can lead to an increase in periodontal temperature. However, in our in vitro study, the simulation of blood flow showed a statistically significant reduction in this temperature increase. This suggests that natural blood flow may mitigate potential thermal effects.

When used correctly, disinfection methods such as heated NaOCl and laser treatments do not appear to have any harmful effects on the surrounding structures in vitro.

These findings suggest that with an appropriate temperature control and technique, the risk of thermal damage can be minimized. However, strict adherence to the treatment protocol is critical to ensure safety and efficacy. Further studies are needed to fully evaluate the long-term effects and to optimize temperature management and antibacterial activity for clinical practice.

## Figures and Tables

**Table 1:** Temperature changes (°C) during disinfection methods representing mean values (L1).

	Flow Rate		
	0 mL/min	2.6 mL/min	7 mL/min
(I) λ445 nm (3 W, cw)			
Median	0.22	1.3	2.35
Minimum	0.00	0.82	1.3
Maximum	0.91	4.33	4.01
Interquartile range	0.67	1.39	0.5
*n*	5	5	5
(II) λ445 nm, 3 W, duty cycle 50%, 10 Hz			
Median	13.11	1.92	1.01
Minimum	5.48	0.00	0.6
Maximum	21.44	6.67	1.41
Interquartile range	14.88	2.06	0.36
*n*	5	5	5
(III) λ445 nm, 3 W, duty cycle 75%, 10 Hz			
Median	21.15	9.57	5.44
Minimum	1.84	4.5	2.86
Maximum	22.83	11.6	11.64
Interquartile range	1.21	1.56	3.83
*n*	5	5	5
(IV) λ970 nm, 2 W, duty cycle 50%, 10 Hz			
Median	0.14	0.87	0.67
Minimum	0.00	0.35	0.19
Maximum	0.52	1.85	1.1
Interquartile range	0.3	0.76	0.31
*n*	5	5	5
(V) λ970 nm, 2 W, duty cycle 75%, 10 Hz			
Median	0.43	2.46	1.17
Minimum	0.00	0.72	0.99
Maximum	7.5	3.91	2.31
Interquartile range	6.53	1.81	0.29
*n*	5	5	5
(VI) PlasmaPen			
Median	3.17	2.44	1.73
Minimum	1.23	0.91	1.58
Maximum	8.28	5.21	5.03
Interquartile range	2.89	1.43	0.65
*n*	5	5	5
(VII) Heatplugger			
Median	14.2	7.52	1.02
Minimum	2.71	6.17	0.65
Maximum	23.1	12.33	1.75
Interquartile range	16.26	1.49	0.5
*n*	5	5	5
(V) Sodium hypochlorite 60 °C			
Median	7.65	2.49	1.45
Minimum	7.06	0.95	0.85
Maximum	10.63	3.68	2.66
Interquartile range	1.93	0.96	1.26
*n*	5	5	5

**Table 2:** Temperature changes (°C) at points P1-3 during the different root canal disinfection techniques.

	P1 (Coronal Third)					P2 (Middle Third)					P3 (Apical Third)				
Groups	Median	Min	Max	IQR	*n*	Median	Min	Max	IQR	*n*	Median	Min	Max	IQR	*n*
Flow rate 0 mL/min															
(I)	0.00	0.00	0.11	0.00	5	0.03	0.00	1.5	0.68	5	3.88	0.22	4.85	2.67	5
(II)	2.65	0.00	7.07	2.79	5	11.82	5.93	22.12	13.02	5	35.52	8.23	63.31	36.63	5
(III)	19.85	9.99	37.98	19.67	5	21.7	20.29	25.02	2.04	5	11.26	7.05	28.21	18.45	5
(IV)	0.00	0.00	1.62	0.02	5	0.35	0.00	0.53	0.42	5	0.44	0.16	3.15	0.97	5
(V)	1.66	0.04	14.26	12.7	5	0.88	0.00	8.34	7.97	5	0.27	0.00	0.61	0.03	5
(VI)	2.66	0.63	5.51	1.49	5	2.7	0.25	8.88	3.59	5	7.47	1.11	10.05	2.79	5
(VII)	25.58	12.01	31.12	16.39	5	18.69	2.98	29.07	17.94	5	0.03	0.00	8.51	5.7	5
(VIII)	9.65	7.31	12.54	2.75	5	7.45	7.00	12.0	0.88	5	7.55	6.31	9.45	1.31	5
Flow rate 2.6 mL/min															
(I)	0.4	0.26	4.1	3.07	5	1.74	1.42	4.53	1.69	5	0.46	0.15	3.92	1.48	5
(II)	4.23	1.8	6.13	0.66	5	2.3	0.00	8.57	3.29	5	0.56	0.00	4.67	0.63	5
(III)	10.94	6.59	16.95	5.44	5	10.6	1.89	14.92	3.29	5	8.06	0.28	10.03	6.85	5
(IV)	2.99	0.54	5.13	2.26	5	0.54	0.19	0.92	0.36	5	0.85	0.05	1.12	0.67	5
(V)	4.29	3.12	4.61	1.48	5	3.39	1.21	5.6	2.8	5	6.76	0.00	11.91	6.76	5
(VI)	6.13	3.21	13.1	2.57	5	2.21	0.94	4.38	2.14	5	0.41	0.17	0.79	0.2	5
(VII)	13.53	12.45	20.28	2.26	5	6.64	4.96	12.56	2.3	5	2.5	1.67	6.08	0.53	5
(VIII)	3.33	2.79	6.1	1.61	5	2.16	0.96	3.78	0.56	5	1.7	0.31	2.24	0.38	5
Flow rate 7 mL/min															
(I)	3.22	1.39	12.7	0.85	5	3.48	1.98	3.86	0.83	5	0.82	0.15	2.02	0.87	5
(II)	1.84	0.9	2.24	0.28	5	1.2	0.52	1.71	0.39	5	1.21	0.81	1.61	0.39	5
(III)	11.28	8.04	29.47	8.29	5	5.3	2.84	13.16	2.34	5	1.62	0.98	2.59	0.21	5
(IV)	1.1	0.75	2.65	0.54	5	0.67	0.2	0.93	0.53	5	1.48	0.4	2.36	1.38	5
(V)	1.15	0.83	2.35	0.61	5	1.17	0.85	2.63	0.78	5	1.87	1.27	3.05	1.2	5
(VI)	2.68	0.95	4.97	1.13	5	1.67	1.2	6.91	0.96	5	1.57	0.1	4.23	0.37	5
(VII)	3.01	2.89	4.96	0.09	5	0.9	0.52	1.48	0.59	5	0.9	0.19	1.57	1.2	5
(VIII)	1.3	8.77	0.69	5.36	5	1.66	1.02	2.44	0.67	5	0.87	0.48	1.19	0.25	5

## Data Availability

The raw data supporting the conclusions of this article will be made available by the authors upon request.
